# Hydrogen Sulfide Reverses Aging-Associated Amygdalar Synaptic Plasticity and Fear Memory Deficits in Rats

**DOI:** 10.3389/fnins.2018.00390

**Published:** 2018-06-07

**Authors:** Jin-Qiong Zhan, Li-Li Zheng, Hai-Bo Chen, Bin Yu, Wei Wang, Ting Wang, Bo Ruan, Bin-Xing Pan, Juan-Ru Chen, Xue-Fen Li, Bo Wei, Yuan-Jian Yang

**Affiliations:** ^1^Biological Psychiatry Laboratory, Department of Psychiatry, Jiangxi Mental Hospital/Affiliated Mental Hospital of Nanchang University, Nanchang, China; ^2^Department of Pharmacy, Jiangxi Maternal and Child Health Hospital, Nanchang, China; ^3^Department of Neurology, Second Affiliated Hospital of Nanchang University, Nanchang, China; ^4^Department of Pharmacology, College of Medical Science, China Three Gorges University, Yichang, China; ^5^Laboratory of Fear and Anxiety Disorders, Institute of Life Science, Nanchang University, Nanchang, China

**Keywords:** aging, hydrogen sulfide, amygdala, synaptic plasticity, fear memory, NMDA receptor

## Abstract

As an endogenous neuromodulator, hydrogen sulfide (H_2_S) exerts multiple biological effects in the brain. Previous studies have shown that H_2_S is involved in the regulation of neural synaptic plasticity and cognition in healthy rodents. It is well known that there is a progressive decline of cognitive function that occurs with increased age. The purpose of this study was to investigate the role of H_2_S in aging-associated amygdalar synaptic plasticity and cued fear memory deficits as well as to explore the underlying mechanisms. We found that H_2_S levels in the amygdala were significantly lower in aged rats when compared with healthy adult rates, which displayed significant deficits in long-term potentiation (LTP) in the thalamo-lateral amygdala (LA) pathway and amygdala-dependent cued fear memory. Bath application of an H_2_S donor, sodium hydrogen sulfide (NaHS), significantly reversed the impaired LTP in brain slices from aged rats, and intra-LA infusion of NaHS restored the cued fear memory in aged rats. Mechanismly, we found that H_2_S treatment significantly enhanced NMDAR-mediated synaptic responses in the thalamo-LA pathway of aged rats. Notably, GluN2B-containing NMDARs, but not GluN2A-containing NMDARs, contributed to the effects of H_2_S on aging-associated impairments of amygdalar LTP and fear memory, because applying GluN2B antagonist could abolish the beneficial effects of NaHS treatment on amygdalar LTP and cognitive performance in aged rats. Collectively, these results show that H_2_S can reverse aging-associated amygdalar synaptic plasticity and fear memory deficits by restoring the function of GluN2B-containing NMDARs, suggesting that supplement of H_2_S might be a therapeutic approach for aging-related cognitive disorders.

## Introduction

Cognitive decline is a natural part of aging (Corey-Bloom et al., 1996; Bishop et al., 2010) and memory is normally the first cognitive domains to decline as individuals age (Singh-Manoux et al., 2012). As a particular kind of memory, fear memory has been shown to be severely affected during the aging process (Deptula et al., 1993; Gould and Feiro, 2005; Fukushima et al., 2008; Kaczorowski et al., 2012; Zeng et al., 2012). Aging-related processes, including inflammatory, oxidative stress, endocrine and immune changes cause functional and anatomical alterations of the amygdala, a key brain area for fear memories, in turn contributing to aging-associated fear memory impairments (McGaugh, 2000; von Bohlen Und Halbach and Unsicker, 2002; Roozendaal et al., 2008; Kumar, 2015).

Synaptic plasticity, which includes long-term potentiation (LTP) and depression (LTD), is a broadly utilized cellular model of memory and learning (Lynch, 2004). Numerous studies have demonstrated a decline in LTP with increased age, and this defect in LTP is believed to underlie age-associated memory impairment (Mothet et al., 2006; Yang et al., 2010; Haxaire et al., 2012). The N-methyl-d-aspartate receptor (NMDAR) is one of the excitatory glutamate receptors known to play a significant role in both memory and learning (Collingridge, 1987; Kumar, 2015). Mounting evidence has indicated that aging is associated with hypofunction of NMDARs in regions of the brain associated with synaptic plasticity, memory, and learning (Kumar, 2015). For instance, NMDAR-mediated excitatory postsynaptic potentials (EPSPs) in the hippocampus are decreased in aged rodents (Yang et al., 2010; Haxaire et al., 2012) and reduced protein expression of NMDARs is observed in the hippocampus of aged animals (Liu et al., 2008; Zhao et al., 2009; Marquez Loza et al., 2017). Nowadays, it is generally accepted that hypofunction of NMDARs contributes to impediments in memory and learning that occur with increased age (Das and Magnusson, 2011; Foster, 2012; Kumar and Foster, 2013; Li et al., 2017) and augmenting the expression and functional activity of the NMDAR subunit could overcome the cognitive impairments in aged animals (Slutsky et al., 2010; Robillard et al., 2011; Brim et al., 2013; Wang et al., 2014).

Hydrogen sulfide (H_2_S) is a highly toxic and flammable gas that is colorless in appearance. Currently, there is mounting evidence to suggest that H_2_S may function as an endogenous gasotransmitter because it regulates several physiological and pathophysiological activities in different biochemical processes (Paul and Snyder, 2015, 2017). There are two mechanisms by which H_2_S produced at high levels (50–160 μmol/L) in the brain, either by the union of cysteine aminotransferase with 3-mercaptopyruvate sulfurtransferase (3-MST) or by the enzyme cystathionine-β-synthase (CBS) (Hu et al., 2011). Abe and Kimura (1996) were the first researchers to investigate the influences of H_2_S on synaptic plasticity and the function of NMDAR function. They showed that physiological concentrations of H_2_S could specifically enhance NMDAR activity and facilitate LTP induction in the hippocampus (Abe and Kimura, 1996). Dysfunction of H_2_S signaling contributes to cognitive impairments in degenerative disorders, such as ischemic cerebral stroke and Alzheimer’s disease (AD) (Li et al., 2011; He et al., 2014; Yang et al., 2016). Aged rats showed decreased hippocampal levels of H_2_S and exogenous H_2_S could alleviate the impaired hippocampal NMDAR-dependent LTP (Li et al., 2017). However, the role that H_2_S may play in aging-associated amygdalar synaptic plasticity and deficits in fear memory remains unknown.

Previously, we have shown that H_2_S can exert a regulatory role in amygdalar LTP and cued fear memory in rats (Wang et al., 2015; Chen et al., 2017). Specifically, treatment with H_2_S promoted cued fear memory and amygdalar LTP by improving NMDAR function in normal rats (Wang et al., 2015); inhibition of endogenous H_2_S generation reduced the synaptic responses of amygdalar NMDARs and impaired cued fear memory in rats (Chen et al., 2017). The aim of this current study was to investigate if H_2_S could reverse aging-associated amygdalar synaptic plasticity and cued fear memory impairments in aged rats. H_2_S levels in the amygdala of aged rats were first examined. Then we investigated the influence of H_2_S donor on aging-associated amygdalar LTP and cued fear memory impairments. Next, NMDAR functions were determined to investigate the mechanisms by which H_2_S exerts beneficial effects on aging.

## Materials and Methods

### Animals

Thirty-two adult (3–4 months) and 60 aged (22–24 months) male Sprague-Dawley rats were obtained from the Hunan SJA Laboratory Animal Company (Changsha, Hunan, China). Rats were fed in a room with controlled light-dark cycle (12:12) and steady temperature (22 ± 2°C). Water and food were supplied *ad libitum*. This research was carried out in accordance with the EU Directive 2010/63/EU and was approved by the Review Committee for the Use of Human or Animal Subjects of Jiangxi Mental Hospital.

### Measurement of H_2_S

The content of H_2_S in amygdala tissue was examined according to a method described in previous studies (Yang et al., 2016; Chen et al., 2017). In brief, the tissue of amygdala was isolated and homogenized in ice-cold KHPO_4_ buffer (pH 7.4, 10 μL buffer per mg tissue). The homogenate was centrifuged, and then 200 μL of supernatant was added to sealed Eppendorf tubes containing 200 μL zinc acetate (1% w/v). Then, 150 μL *N*,*N*-dimethyl-*p*-phenylenediamine sulfate (20 mM) in 7.2 M HCl and 100 μL FeCl_3_ (30 mM) in 1.2 M HCl were added. Reactions were terminated by TCA (10% w/v, 250 μL) after 15 min color development. The resulting solutions were transferred to 96-well plates, and the absorbance of the mixture was measured at 670 nm.

### Electrophysiological Recording

Field potentials recording was used to record LTP and NMDAR-mediated synaptic responses in the thalamo-LA pathway of rats. These procedures were conducted as previously described (Chen et al., 2017). In brief, the brain of the rat was quickly removed, and coronal slices (350 μm) containing amygdala were cut using a vibratome in ice-cold artificial cerebrospinal fluid (ACSF). Slices were recovered for at least 1.5 h by putting them in a holding chamber filled with oxygenated ACSF at 28°C. Then, a single slice was transferred to the perfusion-type recording chamber which was continuously superfused with ACSF pre-gassed with 95% O_2_/5% CO_2_. A bipolar electrode was placed in the internal capsule and a 3.0 M NaCl-filled glass electrode was placed in the LA region to record EPSPs. The stimulation frequency was 0.033 Hz and the stimulating intensity was set to produce an EPSP with 1/3 of the maximal response. High-frequency stimulation (HFS) was used to induce LTP. It consists of five trains at 100 Hz for 1 s, and the interval between trains is 90 s. APV (50 μM), a selective NMDAR antagonist, was used to test whether this LTP was NMDAR-dependent. To isolate NMDAR-mediated synaptic responses, ACSF was changed to magnesium-free ACSF containing glycine (10 μM) and CNQX (10 μM), an AMPA/kainate receptor antagonist (Yang et al., 2016; Chen et al., 2017). 30 μM bicuculline was added into the ACSF to block the activity of GABA_A_ receptors when slices were recorded.

### Fear Conditioning Task

Fear conditioning task was performed according to our previously described method (Chen et al., 2017). One day before fear training, animals were taken into the experimental room and handled for 5-min to make them familiarize the stimuli in the room. On the training day, rats were put into the training chamber for a 3-min acclimatizing period, and then received two pairings of a conditioned stimulus (CS: tone, 80 dB, 30 s) and unconditioned stimulus (US: electric foot shock, 0.75 mA, 1 s). CS and US were co-terminated, and the intertrial interval (ITI) between two trials was 90 s. Rats were stayed in the chamber for 30 s after termination of the procedure and then returned to their cage. 24 h later, cued fear memory was tested. During the test, the rat was placed in a chamber which differentiated from the training chamber for a 3-min acclimatizing period, and then received eight tones (30 s each) with an ITI of 10 s. Freezing was measured as the complete absence of activity except for respiratory movement. Fear memory was assessed by calculating the time spent freezing during the test periods. The freezing behavior was measured by a trained researcher who was blinded from the treatment.

### Measurement of Pain Threshold

The pain threshold was measured according to a method described in our previous study (Chen et al., 2017). After the fear conditioning experiment, the rat was placed into another conditioning chamber for a 3-min acclimatizing period. The electric foot-shocks (1 s) were applied, starting at an intensity of 0.1 mA. The current intensity was increased stepwise by 0.05 mA. The respective current intensity of shock at which the rat began to jump was taken as the pain threshold.

### Open Field Test

The open field test was conducted according to our previously described protocol (Wang et al., 2015). Briefly, an individual rat was allowed to freely behave in an open field arena (40 cm × 40 cm × 40 cm) monitored by a video tracking system. The locomotor activity and the time rats spent in the center region (20 cm × 20 cm) during the 3-min test period were monitored and assessed. The time taken in the central square is used for measuring anxiety-like behaviors.

### Surgery and Injection

The procedures of surgery and injection were performed according to our previous study (Chen et al., 2017). A rat was placed in a stereotaxic apparatus after being anesthetized. Two 22-gauge cannulas were bilaterally implanted in the LA region (+2.8 AP, ±5.0 ML, -8.0 DV from bregma) and secured to the skull with dental acrylic. Rats were recovered for at least 7 days before the behavioral experiments started. When injection was performed, the inner sealing wire was replaced by a 33-gauge injector and drugs were infused into the LA in freely moving rats at a rate of 0.5 μL per min with total volume of 0.5 μL per side. The injector was left for 1.5-min after injection to minimize dragging of injected liquid along the injection track.

### Statistical Analysis

All data are presented as the mean ± SEM and were analyzed using SPSS 18.0 software. The results were statistically analyzed using Student’s *t*-tests or one-way analysis of variance (ANOVA). For ANOVA, *post hoc* comparisons were performed using Bonferroni or Dunnett’s T3 *post hoc* tests, depending on the presence of equal or unequal variance in the groups, respectively. Statistical significance was set at *p* < 0.05.

## Results

### Aged Rats Display Deficits in Amygdalar Synaptic Plasticity and Cued Fear Memory

**FIGURE 1:**
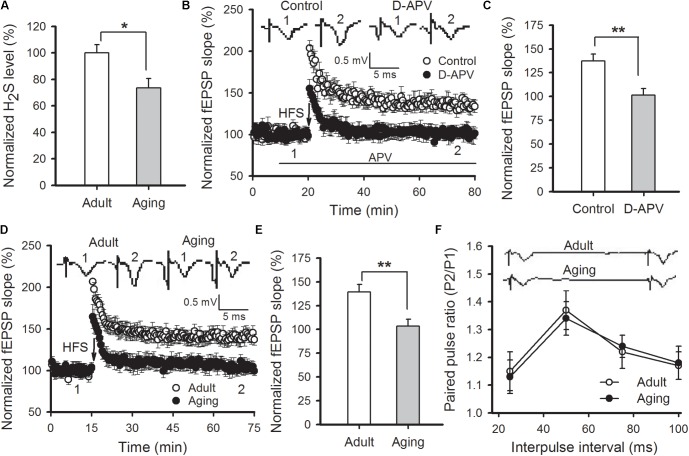
Aged rats display reduced hippocampal H_2_S level and impaired NMDAR-dependent LTP. **(A)** Amygdalar H_2_S levels were lower in aged rats in comparison to healthy adult rats (^∗^*p* < 0.05; *n* = 4 rats per group). **(B)** D-APV (50 μM) effectively blocked HFS-induced LTP in the thalamo-LA pathway of adult rats (*n* = 6–7 slices per group). **(C)** The mean EPSP slope, as depicted through histograms, from 50 to 60 min post-HFS in control and APV-treated slices (^∗∗^*p* < 0.01). **(D)** HFS failed to induce LTP in the thalamo-LA pathway of aged rats (*n* = 8 slices per group). **(E)** The mean EPSP slope, as depicted by histograms, from 50 to 60 min post-HFS in slices obtained from adult and aged rats (^∗∗^*p* < 0.01). **(F)** There was no significant difference in PPF between the healthy adult and aged rats (*n* = 6 slices per group). In **(B,D,F)**, the insets are sample traces from the indicated times on the graph. All data are expressed as the mean ± SEM.

The content of H_2_S in amygdala tissue of aged rats was first measured. We found that amygdalar H_2_S levels in aged rats were lower than those detected in adult rats (*p* < 0.05; **Figure 1A**). H_2_S is a modulator for NMDAR function. We then adopted field potentials recording to examine NMDAR-dependent LTP in the thalamo-LA pathway of aged rats. HFS evoked a stable LTP in the thalamo-LA pathway from the adult rat slices (137.3 ± 7.1% of baseline), while pre-incubation with D-APV (50 μM), a NMDAR antagonist, for 10 min obviously blocked the LTP induction (101.4 ± 7.0% of baseline; *p* < 0.01 vs. control; *n* = 6–7 slices per group), suggesting that the LTP evoked by HFS was NMDAR-dependent (**Figures 1B,C**). In agreement with a previous study (Zeng et al., 2012), a significant suppression of NMDAR-dependent LTP was observed in the thalamo-LA pathway of aged rats in this study (adult rat 139.2 ± 7.9%, aged rat 105.4 ± 7.3%; *p* < 0.01; *n* = 8 slices per group; **Figures 1D,E**). Paired-pulse facilitation (PPF) is a common indicator used for evaluating presynaptic function. There was no significant difference in PPF between the two groups (*p* > 0.05; **Figure 1F**), suggesting that impairment of amygdalar LTP in aged rats should attribute to a modification in postsynaptic responsiveness, but not be from the altered release of the presynaptic neurotransmitter.

**FIGURE 2:**
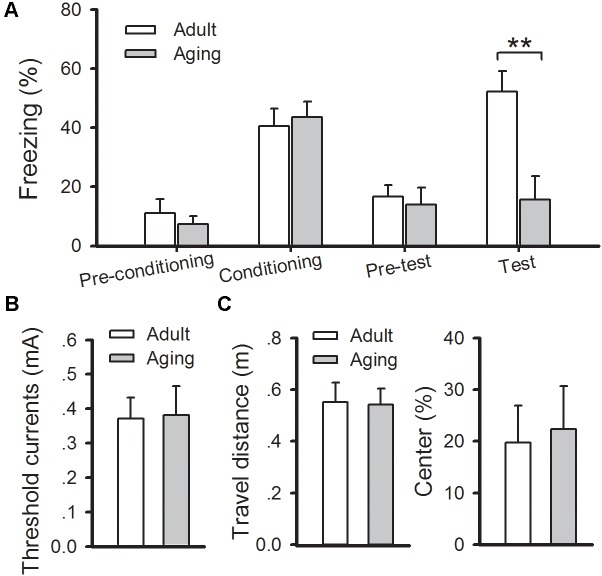
Aged rats exhibit a deficit in cued fear memory. **(A)** During fear training, there was no observable difference in freezing rates between healthy adult and aged rats, while freezing responses were significantly decreased in aged rats during cued memory test (^∗∗^*p* < 0.01). **(B)** There was no significant difference in the pain threshold between the two groups. **(C)** There was no significant difference in either locomotion (left) or anxiety (right) between the two groups. *N* = 7 rats per group. All data are expressed as the mean ± SEM.

Then classical fear conditioning paradigm was conducted to evaluate changes in amygdala-dependent memory in aged rats. On the day of conditioning, rats were given two-tone (CS) – footshock (US). The aged rats displayed normal acquisition during the training phase, and a similar number of freezing responses was observed in aged rats compared to the adults (*p* > 0.05). At 24 h post-conditioning, the rats were put into a new chamber and the auditory conditioned stimulus tone was delivered 3 min later. Significantly increased levels of freezing elicited by the tone were observed in the adult rats, whereas the aged rats displayed a significant reduction in the number of freezing responses when compared with the adult rats (*p* < 0.01) (**Figure 2A**). Notably, the reduction in freezing behavior in aged rats was not due to adjustments in their pain threshold, state of anxiety, or locomotion of animals because no difference in these indexes was found between the two groups (**Figures 2B,C**). Altogether, our results confirm that aged rats exhibited deficits in cued fear memory and amygdalar NMDAR-dependent LTP.

### H_2_S Donor Reverses the Impaired Amygdalar LTP and Fear Memory in Aged Rats

**FIGURE 3:**
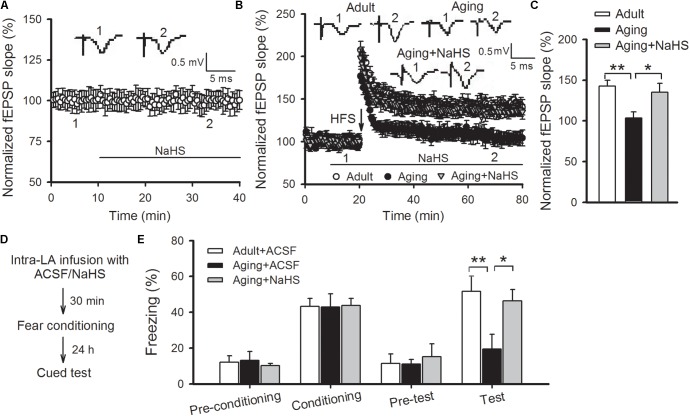
NaHS reverses the impaired amygdalar LTP and cued fear memory in aged rats. **(A)** Bath-applied NaHS (75 μM) had no effects on the basal EPSPs recorded in the thalamo-LA pathway of aged rats (*n* = 8 slices). **(B)** NaHS restored LTP in the thalamo-LA pathway in aged rats (*n* = 6–9 slices per group). **(C)** The mean EPSP slope, as illustrated by histograms, from 50 to 60 min post-HFS (^∗^*p* < 0.05, ^∗∗^*p* < 0.01). **(D)** Schematic describing the behavioral experiment setup. **(E)** Freezing responses were unaffected by intra-LA infusion with NaHS (75 μM, 0.5 μL per side) in aged rats during fear conditioning; however, the freezing rate was increased to levels consistently found in ACSF-treated adult rats during cued memory testing (^∗^*p* < 0.05, ^∗∗^*p* < 0.01; *n* = 5–8 rats per group). All data are expressed by mean ± SEM.

Next, we investigated whether application of an H_2_S donor could undo the amygdalar LTP and cued fear memory impairments in aged rats. NaHS is a commonly utilized exogenous donor for H_2_S. Exposure to 75 μM NaHS via bath application in slices of aged rats did not affect the basal neurotransmission (**Figure 3A**), but significantly enhanced the amygdalar NMDAR-dependent LTP to a level commonly found in adult rats (*p* < 0.05 vs. aging control; *n* = 6–9 slices per group; **Figures 3B,C**). In accordance with the electrophysiological findings, intra-LA infusion of 0.5 μL of 75 μM NaHS (per side) in aged rats 30 min prior to fear conditioning improved the freezing rate to that found in healthy adult rats [ANOVA, *F*_(2,18)_ = 5.130, *p* = 0.017; *n* = 6–9 rats per group; **Figures 3D,E**]. As previously reported (Wang et al., 2015), treatment with NaHS did not significantly impact anxiety levels, sensitivity to pain, or locomotion in aged rats, ruling out the possibility that the effect of NaHS in aged rats was a gross change in pain sensitivity or anxiety (data not shown). Statistical analyses revealed a significant difference in mean freezing rates between ACSF-treated aged rats and NaHS-treated aged rats (*p* = 0.048), and the freezing rate of the NaHS-treated aged rats was indistinguishable from the level of the adult group (*p* > 0.05). These data suggest that the application of H_2_S may undo impaired amygdalar LTP and cued fear memory in aged rats.

### H_2_S Donor Improves Amygdalar GluN2B-Containing NMDAR Function in Aged Rats

**FIGURE 4:**
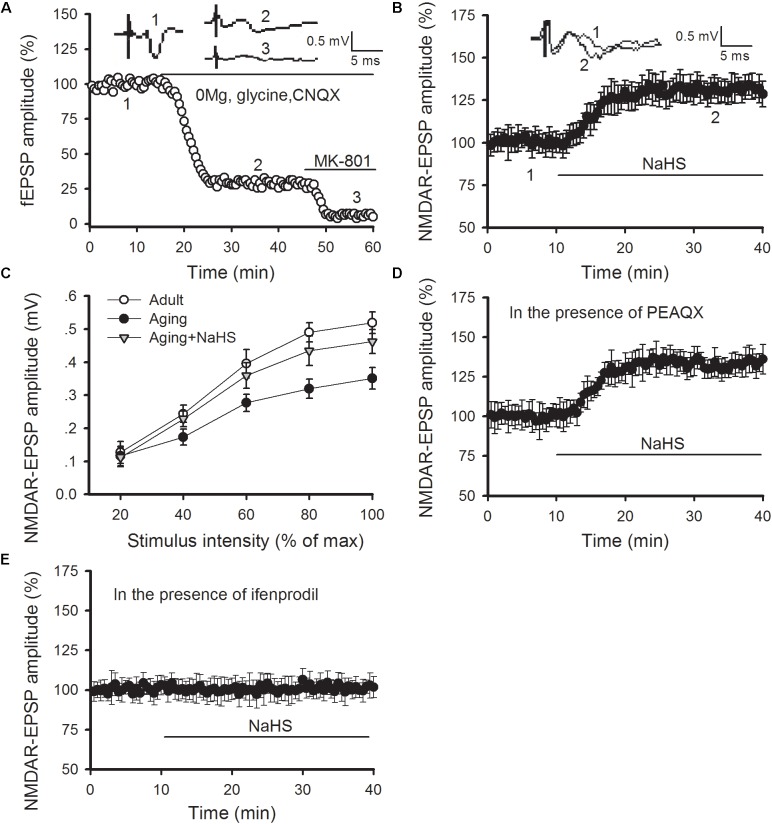
NaHS restores amygdalar NMDAR-mediated synaptic responses by regulating GluN2B-containing NMDAR function in aged rats. **(A)** NMDAR-mediated EPSPs in the thalamo-LA pathway were isolated by pharmacological intervention. Treatment with MK-801, an NMDAR non-competitive inhibitor, showed complete inhibition of EPSPs. **(B)** NaHS (75 μM) in slices obtained from aged rats increased the amplitude of NMDAR-EPSPs in the thalamo-LA pathway (*n* = 7 slices). **(C)** As shown by input–output curves, NaHS increased the amplitude of NMDAR-EPSPs to that of healthy adult controls in the slices from aged rats (*p* < 0.05 vs. aging alone) (*n* = 5 slices per group). **(D)** PEAQX (0.4 μM) pre-treatment did not impact the effect of NaHS on NMDAR-mediated EPSPs in aged rats (*n* = 6 slices). **(E)** Ifenprodil (3 μM) pre-treatment inhibited the action of NaHS on NMDAR-mediated EPSPs in aged rats (*n* = 6 slices). All data are expressed by mean ± SEM.

Previous studies have revealed that H_2_S can regulate NMDAR function in LA neurons as well as amygdalar synaptic plasticity and cued fear memory (Wang et al., 2015; Chen et al., 2017). Hence, we tested whether the benefits of H_2_S in LTP and fear memory in aged rats arose from its regulatory role in NMDAR function. The NMDAR-mediated synaptic potentials were isolated by exchanging the normal ACSF for Mg^2+^-free ACSF, which contained 10 μM glycine and 10 μM of an AMPA receptor antagonist known as CNQX (**Figure 4A**). We found that NaHS treatment (75 μM) increased the amplitude of NMDAR-mediated EPSPs in the thalamo-LA synapses of aged rats [ANOVA, main effect of NaHS *F*_(1,6)_ = 333.416, *p* < 0.001; **Figure 4B**]. Then input-output curves for NMDAR-EPSPs were conducted to further evaluate the effect of H_2_S on NMDAR function in aged rats. The amplitude of NMDAR-mediated EPSPs increased with incremental stimulus intensity (**Figure 4C**); however, the mean amplitude of NMDAR-mediated EPSP recorded in the slices obtained from aged rats was significantly lower than that of the healthy adult controls when the same stimulation was delivered. Also, bath application of NaHS in slices from aged rats increased the NMDAR-mediated EPSP to a level that was comparable to that of adult rats (*n* = 5 slices per group). The ANOVA (mixed model) for input-output curves showed a significant aging main effect [*F*_(1,9)_ = 18.219, *p* < 0.001] and a significant NaHS treatment effect [*F*_(1,9)_ = 9.720, *p* = 0.003], suggesting that administration of H_2_S could rescue amygdalar NMDAR function in aged rats.

We further explored the influence of H_2_S on GluN2A- and GluN2B-containing NMDAR function by pharmacologic manipulation. PEAQX is a specific antagonist of GluN2A-containing NMDARs. In the presence of PEAQX (0.4 μM), NaHS treatment could still increase the NMDAR-EPSPs recorded in the thalamo-LA synapses of aged rats [ANOVA, main effect of NaHS *F*_(1,5)_ = 374.676, *p* < 0.001; **Figure 4D**]. However, NaHS treatment failed to cause an increase in NMDAR-EPSPs in the thalamo-LA pathway in the presence of ifenprodil (3 μM), which is a specific antagonist of GluN2B subunit (**Figure 4E**). Together, these findings indicate that the GluN2B subunit is likely involved in the mechanism by which H_2_S effects amygdalar NMDAR function in aged rats.

### Blockade of GluN2B Abolishes the Beneficial Effects of H_2_S on Amygdalar LTP and Fear Memory in Aged Rats

**FIGURE 5:**
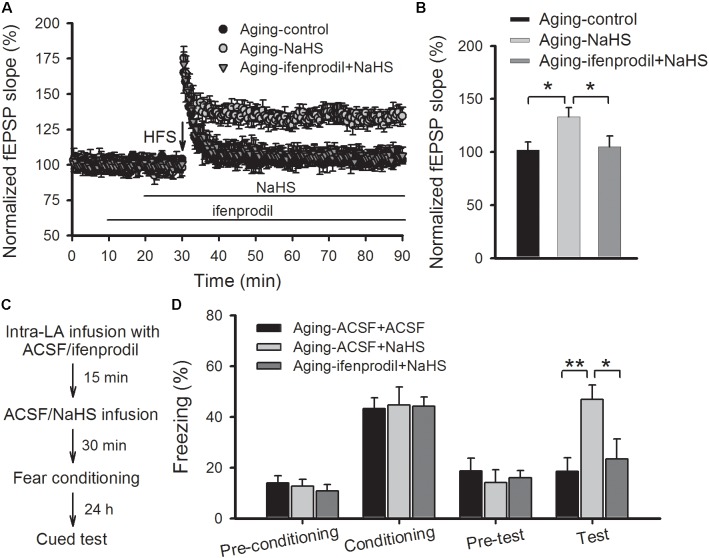
Treatment with a GluN2B antagonist abolishes the benefits of NaHS treatment on amygdalar LTP and fear memory in aged rats. **(A)** Pre-treatment with ifenprodil (3 μM) eliminated the benefits of NaHS on NMDAR-dependent LTP in the thalamo-LA pathway of aged rats (*n* = 6–8 slices per group). **(B)** The mean EPSP, as illustrated by histograms, from 50 to 60 min post-HFS (^∗^*p* < 0.05). **(C)** Schematic describing the behavioral experiment setup. **(D)** The mean freezing rate of rats during fear training and memory test sessions. Treatment with ifenprodil (0.2 μg, 0.5 μL per side) has no effect on behavioral performance in the training session but abolished the improvements, that resulted from NaHS treatment, on cued fear memory in aged rats (^∗^*p* < 0.05, ^∗∗^*p* < 0.01; *n* = 5–7 rats per group). All data are expressed by mean ± SEM.

We then investigated whether blocking GluN2B-containing NMDARs modulated the beneficial effects of H_2_S in aged rats. As shown in **Figures 5A,B**, pretreating slices of aged rats with ifenprodil (3 μM) observably abolished the rescued effect of NaHS on the induction of amygdalar LTP (*p* < 0.05 vs. NaHS treatment alone; *n* = 6–8 slices per group). In a separate set of experiments, aged rats were bilateral intra-LA infused with ifenprodil or ACSF (0.2 μg per side) at 15 prior to NaHS infusion, and 30 min following NaHS treatment, were fear conditioned (**Figure 5C**). We found that treatment with ifenprodil in aged rats did not affect the behavioral performance during the training session, yet it observably blocked the enhancement effect of NaHS on cued fear memory [ANOVA, *F*_(2,16)_ = 7.848, *p* = 0.005; **Figure 5D**]. *Post hoc* comparisons using Bonferroni’s test revealed that the freezing level in the NaHS-treated aged rats that were pre-infused with ifenprodil was not different from the freezing rate in ACSF-treated aged rats during fear memory test (*p* > 0.05), indicating that the GluN2B subunit may mediate the beneficial effects of H_2_S in aged rats.

## Discussion

In this study, our primary finding was that supplementing aged rats with H_2_S can reverse deficits in cued fear memory and amygdalar NMDAR-dependent LTP, which is a validated model of memory and learning. The H_2_S effects may be associated with the upregulation of GluN2B-containing NMDAR because treatment with NaHS enhanced the synaptic responsiveness of GluN2B-containing NMDARs in the thalamo-LA pathway of aged rats and a GluN2B-specific antagonist could abolish the benefits of H_2_S in amygdalar LTP and cognitive performance. These findings suggest that H_2_S supplementation may be a promising strategy for the treatment of age-related fear memory deficits.

H_2_S acts as an endogenous neuromodulator in the brain (Hu et al., 2011). H_2_S at physiological concentration can promote NMDAR-dependent LTP, which is correlated with behaviorally relevant memory functions, in both amygdala and hippocampus (Abe and Kimura, 1996; Wang et al., 2015). An increase of H_2_S signal in the limbic system *in vivo* can improve fear memory in aged rats (Wang et al., 2015; Li et al., 2017). Accumulating studies have demonstrated a link between dysfunction of H_2_S signaling and the pathogenesis of age-related neurodegenerative disorders, including Parkinson’s disease (PD), AD and cerebral ischemia (Hu et al., 2011; Li et al., 2011; Yang et al., 2016). For instance, decreased H_2_S levels have been noted in the brains of AD animal models (He et al., 2014; Yang et al., 2016) and administration of H_2_S can alleviate the neuropathophysiological changes and undo the deficits in cognitive and synaptic plasticity in AD animal models (Giuliani et al., 2013; He et al., 2014; Yang et al., 2016). In this study, we demonstrate that amygdalar H_2_S levels were significantly decreased in aged rats and NaHS treatment could reverse the impairments in amygdalar NMDAR-dependent LTP and cued fear memory, suggesting that endogenous H_2_S has an important influence in aging-associated fear memory deficits.

What are the molecular mechanisms underlying the beneficial role that H_2_S plays in the synaptic and cognitive impairments of aged rats? NMDAR is a known action target of H_2_S in neurons. H_2_S could improve NMDAR-mediated synaptic responses in both amygdala and hippocampus (Abe and Kimura, 1996; Wang et al., 2015). Previously, NMDAR function was found to be impaired when endogenous H_2_S levels were suppressed (Chen et al., 2017). Hypofunction of NMDARs was shown to contribute to the cognitive impairments resulting from aging and many aging-related neurodegenerative diseases (Barnes et al., 1997; Kumar and Foster, 2013; Yang et al., 2016). Therefore, we surmise that the benefits of H_2_S in aged rats arise from its regulatory role in amygdalar NMDARs. This assumption was tested by recording NMDAR-mediated synaptic responses (Chen et al., 2017). The input-output responses of NMDAR-mediated potentials in the thalamo-LA pathway of aged rats were much lower than those of adult rats. In slices obtained from aged rats, application with H_2_S donor could observably increase NMDAR-mediated EPSP amplitude and return the NMDAR-EPSPs to levels found in healthy adult rats. The expression of GluN2A and GluN2B, two essential modulatory subunits of NMDAR, was decreased in multiple brain regions of aged rodents (Magnusson et al., 2007; Liu et al., 2008; Zhao et al., 2009). In this study, we showed that H_2_S donor could increase NMDAR-mediated synaptic potentials in the thalamo-LA synapses of aged rats with the presence of GluN2A specific antagonist, whereas no increase was observed in NMDAR-mediated EPSPs with the presence of GluN2B antagonist, suggesting that H_2_S specifically regulated the activity of GluN2B-containing NMDARs in the amygdala of aged rats. Furthermore, antagonizing GluN2B could abolish the benefits of H_2_S in the impairments of amygdalar LTP and cued fear memory in aged rats, suggesting that GluN2B-containing NMDARs may be a critical molecular target of H_2_S action.

GluN2B-containing NMDARs are proposed to mainly localize on the extrasynaptic site and considered as a crucial factor involved in apoptosis (Hardingham and Bading, 2010; Zhou et al., 2015). However, there is growing evidence suggesting that GluN2B-containing NMDARs may be functional at the synapse of the amygdala (Lopez de Armentia and Sah, 2003; Miwa et al., 2008; Duan et al., 2015). For instance, blockade of the receptors leads to deficits in amygdala-dependent LTP and memory (Rodrigues et al., 2001; Muller et al., 2009) and enhancement of the receptors promotes amygdalar synaptic plasticity and fear memory in rats (Abumaria et al., 2011; Wang et al., 2015). The GluN2B subunit is severely affected by the aging process (Magnusson et al., 2007; Zhao et al., 2009). Our results show that H_2_S treatment selectively enhanced GluN2B-mediated synaptic responses in the thalamo-LA pathway of aged rats and specific GluN2B antagonist abolished the benefits of H_2_S donor in amygdalar NMDAR-dependent cued fear memory and LTP, not only indicate that upregulation of GluN2B function is responsible for the effects of H_2_S in aged rats but also suggest that hypofunction of GluN2B-containing NMDARs contribute to aging-associated amygdalar synaptic plasticity and cognitive defects. However, the mechanisms by which GluN2B subunits are regulated by the H_2_S in the amygdala requires further investigations.

Activation of GluN2B and GluN2A is thought to be needed for hippocampal LTD and LTP, respectively (Liu et al., 2004). However, mounting evidence has indicated that GluN2B subunit is required for the induction of amygdalar LTP (Miwa et al., 2008; Muller et al., 2009; Wang et al., 2015). Perhaps the differences in biophysical properties and protein expression of the two subunits between amygdala and hippocampus could be responsible for this discrepancy. Specifically, more GluN2B subunits were found in the synapses of LA neurons compare to the CA1 region (Lopez de Armentia and Sah, 2003). The ratio of NMDA-EPSCs to AMPA-EPSCs in the LA neurons is significantly larger than that in the CA1 region and the effect of Mg^2+^ on the NMDARs is much lower in the LA neurons than in the CA1 neurons (Miwa et al., 2008). Our previous study showed that improvements in amygdalar LTP and emotional memory by H_2_S treatment were dependent on the activation of GluN2B-containing NMDARs (Wang et al., 2015). In the study, the restoration of GluN2B-containing NMDARs is shown to contribute to the beneficial properties of H_2_S on amygdalar LTP and fear memory in aged rats, also providing evidence for the importance of GluN2B subunit in amygdalar synaptic plasticity and memory.

There are some limitations in this study. First, due to the technological difficulties of performing whole-cell patch clamp recording in brain neurons of aged animals, we adopted field potential recording to detect a NMDAR-mediated synaptic response in the thalamo-LA synapses by pharmacologic manipulation. In the future, whole-cell patch clamp recording may allow for the direct measurement of NMDAR-mediated excitatory postsynaptic current in LA neurons to confirm the influence of H_2_S on NMDAR function in aged rats. Second, a single concentration and dosage of NaHS, selected from our previous studies (Wang et al., 2015; Chen et al., 2017), was used to investigate the influence of H_2_S on the impaired fear memory and synaptic plasticity in the amygdala. In the future, other concentration and dosage should be investigated to explore the effective range of H_2_S in the treatment of aging-associated amygdalar LTP and fear memory deficits. Moreover, whether the beneficial effects of H_2_S found in aged rats could be generalized to other animal species, especially for human being remains unknown. In addition, considering the lethality of H_2_S at high concentrations, determining the lowest effective dose will help with treatment planning. H_2_S could enhance the excitotoxicity of glutamate after a stroke and aggravate seizure-like events (Qu et al., 2006; Luo et al., 2014). Thus, the potential risks of brain damage and epileptic seizures should also be assessed during treatment planning.

## Conclusion

We show that H_2_S can reverse amygdalar NMDAR-dependent LTP and fear memory deficits in aged rats, and the mechanism might be correlated to upregulation of GluN2B-containing NMDAR function. H_2_S is a gaseous molecule that can rapidly cross the blood-brain barrier. Inhaled H_2_S has been shown to produce a similar cognitive regulatory effect to that of the amygdala-specific delivery of the H_2_S donor (Wang et al., 2015). Thus, H_2_S inhalation may be an excellent therapy for synaptic plasticity and cognitive impairments in the elderly population.

## Author Contributions

Y-JY and BW wrote the paper and designed the research. J-QZ, L-LZ, H-BC, WW, BR, J-RC, and X-FL performed the research. BY, TW, B-XP, and Y-JY analyzed the data.

## Conflict of Interest Statement

The authors declare that the research was conducted in the absence of any commercial or financial relationships that could be construed as a potential conflict of interest.
